# ADAM2 Interactions with Mouse Eggs and Cell Lines Expressing α_4_/α_9_ (ITGA4/ITGA9) Integrins: Implications for Integrin-Based Adhesion and Fertilization

**DOI:** 10.1371/journal.pone.0013744

**Published:** 2010-10-29

**Authors:** Ulyana V. Desiderio, Xiaoling Zhu, Janice P. Evans

**Affiliations:** Department of Biochemistry and Molecular Biology, Bloomberg School of Public Health, Johns Hopkins University, Baltimore, Maryland, United States of America; Ottawa Hospital Research Institute and University of Ottawa, Canada

## Abstract

**Background:**

Integrins are heterodimeric cell adhesion molecules, with 18 α (ITGA) and eight β (ITGB) subunits forming 24 heterodimers classified into five families. Certain integrins, especially the α_4_/α_9_ (ITGA4/ITGA9) family, interact with members of the ADAM (a disintegrin and metalloprotease) family. ADAM2 is among the better characterized and also of interest because of its role in sperm function. Having shown that ITGA9 on mouse eggs participates in mouse sperm-egg interactions, we sought to characterize ITGA4/ITGA9-ADAM2 interactions.

**Methodology/Principal Findings:**

An anti-β_1_/ITGB1 function-blocking antibody that reduces sperm-egg binding significantly inhibited ADAM2 binding to mouse eggs. Analysis of integrin subunit expression indicates that mouse eggs could express at least ten different integrins, five in the RGD-binding family, two in the laminin-binding family, two in the collagen-binding family, and ITGA9-ITGB1. Adhesion assays to characterize ADAM2 interactions with ITGA4/ITGA9 family members produced the surprising result that RPMI 8866 cell adhesion to ADAM2 was inhibited by an anti-ITGA9 antibody, noteworthy because ITGA9 has only been reported to dimerize with ITGB1, and RPMI 8866 cells lack detectable ITGB1. Antibody and siRNA studies demonstrate that ITGB7 is the β subunit contributing to RPMI 8866 adhesion to ADAM2.

**Conclusions/Significance:**

These data indicate that a novel integrin α-β combination, ITGA9-ITGB7 (α_9_β_7_), in RPMI 8866 cells functions as a binding partner for ADAM2. ITGA9 had previously only been reported to dimerize with ITGB1. Although ITGA9-ITGB7 is unlikely to be a widely expressed integrin and appears to be the result of “compensatory dimerization” occurring in the context of little/no ITGB1 expression, the data indicate that ITGA9-ITGB7 functions as an ADAM binding partner in certain cellular contexts, with implications for mammalian fertilization and integrin function.

## Introduction

Integrins are a family of cell adhesion molecules that mediate cell-cell and cell-extracellular matrix interactions [1], [2], [3] that also have been implicated as having a role in contributing to mammalian sperm-egg interactions. Integrins are heterodimeric transmembrane proteins made of an α and a β subunit, with 18 α and eight β subunits in mammals. (Note: Traditional nomenclature refers to α and β subunits; Human Genome Organization- and Mouse Genome Database-approved nomenclature refers these as ITGA and ITGB subunits respectively, and we will use that terminology here. For example, α_9_ is ITGA9 and β_1_ is ITGB1.) The 24 known heterodimer combinations are classified into five different subfamilies based sequence homologies of the α subunits and on ligand specificity [1], [4], [5]. One of the more recently characterized groups of integrin ligands is the ADAM (a disintegrin and metalloprotease) family of proteins, with the adhesive activity largely mediated by the disintegrin-like domain, so-named due to the domain's homology to disintegrin domains in snake venom metalloproteases that interact with integrins [6], [7], [8], [9].

This study examines α_4_/α_9_ (ITGA4/ITGA9) integrin interactions with ADAM2 in the context of mammalian sperm-egg interactions and also general cell adhesion. We have demonstrated that an egg ITGA4/ITGA9 integrin functions in murine fertilization; RNAi-mediated knockdown of *Itga9* in mouse eggs reduces sperm binding and subsequent fusion [10] and treatment of mouse eggs with a specific peptide inhibitor of ITGA4/ITGA9 integrins reduces the binding of recombinant ADAM2 [11]. Mouse and human eggs express ITGA9 [10], and ITGA4 expression has been observed in bovine, hamster, pig, and human eggs [12], [13], [14], [15]. Both ITGA4 and ITGA9 dimerize with ITGB1, and ITGA4 also dimerizes with ITGB7. ITGB1 is expressed by eggs in numerous mammalian species [12], [14], [15], [16], [17], [18], [19], [20]. ITGB1 is not essential for fertilization in mice based on studies of an oocyte-specific *Itgb1* conditional knockout [21], although *Itgb1*-deficient eggs do have subtle defects, as recent studies have revealed delayed sperm binding to *Itgb1*-deficient eggs [22], in agreement with observations of reduced sperm binding to eggs treated with anti-ITGB1 antibodies [10], [14], [19], [23].

ADAM2 is one of the ADAM family members on sperm and is one of the best characterized ADAMs in terms of its interactions with cells and with integrins [23], [24], [25], [26], [27]. Studies of *Adam2*-null mice reveal defects in several sperm functions, including sperm-egg binding [28], [29], [30], [31], [32]. The adhesion-mediating motif of ADAM2 is well-conserved among the ADAMs, making it a good “model” ADAM for ADAM-integrin interactions. ADAM2 has an ECD tripeptide in the disintegrin domain that was characterized by structure-function studies as important for ADAM2 binding to eggs [24], [25], and also has a sequence similar to the RX_6_DLPEF motif in ADAM15 that is important in binding ITGA9-ITGB1 [33] (RLAQDECDVTEY in mouse ADAM2, RPSFEECDLPEY in human ADAM2). Additionally, three of the seven residues in ADAM28 implicated in interaction with ITGA4-ITGB1 [34] are conserved in ADAM2.

We sought to characterize ITGA4/ITGA9 function as an ADAM2 binding partner through assays of ADAM2 disintegrin domain interactions with mouse eggs and also through cell adhesion assays with cell lines known to express each of the ITGA4/ITGA9 integrins. As noted above, the ITGA4/ITGA9 integrin family has three known members: ITGA4-ITGB1, ITGA4-ITGB7, and ITGA9-ITGB1. Experiments here first examined mouse eggs, addressing the question of whether ITGB1 (as the partner to ITGA9) is the primary integrin in mouse eggs that mediates egg interactions with ADAM2. Second, since ITGA4 expression has been observed in bovine, hamster, pig, and human eggs [12], [13], [14], [15], we examined the interactions of the ITGA4 integrins, ITGA4-ITGB1 and ITGA4-ITGB7, with ADAM2. These experiments produced an unexpected and intriguing result. These studies used RPMI 8866 cells, a human B-cell lymphoblastoid cell line that is commonly used to study ITGA4-ITGB7-mediated cell adhesion; these cells express little or no ITGB1 on the cell surface [35], [36], [37], [38], [39]. These cell adhesion assays demonstrated that RPMI 8866 cells adhere to ADAM2, but a surprise came from assays using function-blocking antibodies that revealed that this adhesion was inhibited not only by anti-ITGA4 antibodies but also an anti-ITGA9 antibody. This was of significant interest because ITGA9 has only been reported to dimerize with ITGB1, and as noted, RPMI 8866 cells have low to undetectable levels of ITGB1, in agreement with the fact that anti-ITGB1 antibodies have no effect on RPMI 8866 adhesion to multiple substrates, including ADAMs [36], [39]. Our siRNA and function-blocking antibody studies demonstrate that ITGB7 is the β subunit that contributes to adhesion to ADAM2. These studies provide new insights into ADAM-integrin interactions and into integrin biology, suggesting the presence of a new integrin heterodimer, ITGA9-ITGB7, in RPMI 8866 cells that can interact with ADAM2.

## Results

**Figure 1:**
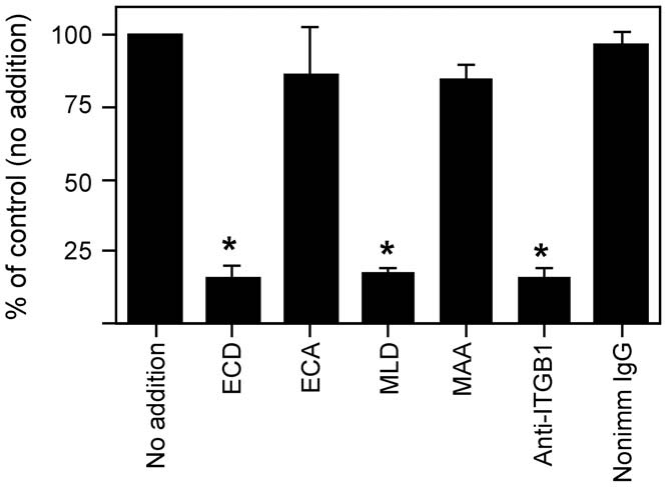
ADAM2 binding to eggs. Eggs were left untreated (no addition), or treated with the indicated peptide (100 µM; ECD peptide, corresponding to the ADAM2 disintegrin loop; its negative control ECA; the ITGA4/ITGA9-blocking peptide MLD, or its control MAA) or 100 µg/ml of the function-blocking anti-ITGB1 monoclonal antibody Hmβ1-1 antibody (anti-β_1_) or Armenian hamster nonimmune IgG (Nonimm IgG). Data were normalized to the amount of egg-associated ADAM2 with the “no addition” controls. Asterisks indicate p<0.05 as compared to untreated controls and the appropriate control peptide or nonimmune antibody.

As noted above, our work implicates an ITGA4/ITGA9 integrin on eggs in sperm-egg adhesion [10], [11]. We also have shown that the anti-mouse ITGB1 function-blocking antibody Hmβ1-1 reduces sperm-egg binding [10], and therefore we examined the effect of this Hmβ1-1 anti-ITGB1 antibody on the binding of ADAM2 to eggs, comparing its effects side-by-side with the effects of two control reagents: an ECD peptide, corresponding to the ECD-containing adhesion-mediating motif in the ADAM2 disintegrin domain [24], and an MLD peptide, a peptide that blocks ITGA4/ITGA9 integrins [11], [40], [41], [42]. Eggs treated with the control reagents (point-mutated peptides ECA and MAA, for ECD and MLD respectively; or nonimmune antibody) had levels of ADAM2 binding that were comparable to untreated eggs (Fig. 1). The extent of the anti-ITGB1 antibody-mediated inhibition was similar to the extent of inhibition observed with the positive control reagents, the ECD and MLD peptides (Fig. 1), suggesting that ITGB1 integrins are the major integrins contributing to ADAM2 interaction with the egg plasma membrane.

**Figure 2:**
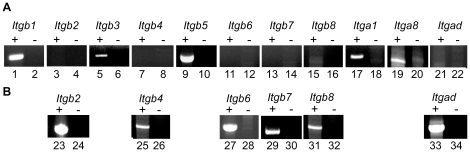
Integrin subunit mRNAs in mouse oocytes. RT-PCR analysis of *Itgb* subunit and selected *Itga* subunit expression in mouse oocytes (Panel A; lanes 1–22) or control tissues (Panel B; spleen, lanes 23–28, 33–34; liver, lanes 29–30). First strand cDNA was prepared from RNA with reverse transcriptase (+; odd-numbered lanes), or, as a negative control, without RT (−; even-numbered lanes). This shows that oocytes express *Itgb1*, *Itgb3*, *Itgb5*, *Itga1*, and *Itga8* mRNA. Although *Itgb2*, *Itgb4*, *Itgb6*, *Itgb7*, *Itgb8*, and *Itgad* were not detected in oocytes, these PCR products could be amplified from control cDNA.

To complement this, we sought a complete picture of β subunit expression in mouse eggs; we also included examination three α integrin subunits (*Itga1*/α_1_, *Itga8*/α_8_, *Itgad*/α_D_) whose expression has not yet been confirmed in mouse oocytes but had been implicated from transcriptome analyses ([43], Unigene). Although several of these integrin subunits have been shown to have limited tissue distribution, there is precedent for oocytes expressing a variety of members of other molecular families that had previously been thought to have very restricted expression [44]. This RT-PCR analysis showed that mouse oocytes express *Itgb1*, *Itgb3*, *Itgb5*, *Itga1*, and *Itga8* (Fig. 2). No PCR products were amplified from control oocyte cDNA preparations from which the reverse transcriptase had been omitted, indicating that the PCR products were amplified from cDNA (Fig. 2). We did not detect *Itgb2*, *Itgb4*, *Itgb6*, *Itgb7*, *Itgb8*, or *Itgad* in mouse oocytes (Fig. 2), while we could detect these transcripts in control tissues (spleen or liver as indicated; Fig. 2).

We also sought to characterize cellular interactions with ADAM2 in cell lines expressing the three known members of the ITGA4/ITGA9 family (ITGA4-ITGB1, ITGA9-ITGB1, ITGA4-ITGB7) as a complementary part of this work, since ITGA4 expression has been reported for bovine, hamster, pig, and human eggs, and there is some evidence for ITGB7 expression by human eggs [12], [13], [14], [15]. Human cell lines are particularly useful for studies of integrin-mediated adhesion because of the wealth of reagents, such as specific function-blocking monoclonal antibodies. (For example, the anti- ITGA9 Y9A2 monoclonal antibody does not label mouse eggs by immunofluorescence [data not shown], and thus could not be used for studies of sperm or ADAM2 binding to mouse eggs.)

**Figure 3:**
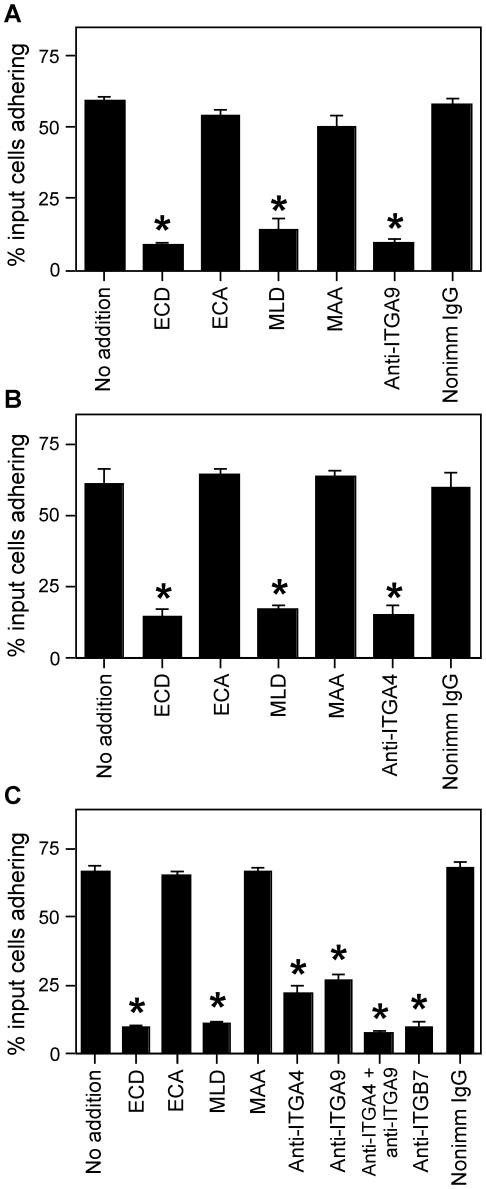
ITGA4/ITGA9-mediated cell adhesion to ADAM2. Cell adhesion assays were performed with the indicated cell lines (Panel A, Tera-2 cells; Panel B, HT1080 cells; Panel C, RPMI 8866 cells) with 50 µg/ml of recombinant ADAM2 as substrate. Cells were left untreated (no addition), or treated with the indicated peptide (100 µM; ECD peptide, corresponding to the ADAM2 disintegrin loop; its negative control ECA; the ITGA4/ITGA9-blocking peptide MLD, or its control MAA) or the indicated antibody (20 µg/ml; function-blocking anti-ITGA9 monoclonal antibody Y9A2 [Panels A and C]; the function-blocking anti-ITGA4 monoclonal antibody PS-2 [Panels B and C]; function-blocking anti-ITGB7 monoclonal antibody FIB27 [Panel C]; or a species-matched nonimmune IgG). The y-axes indicate the percentage of the input cells left adherent after washing; errors bars represent the SEM. Asterisks indicate p<0.05 as compared to untreated controls and the appropriate control peptide or nonimmune antibody.

These studies examined cell adhesion to ADAM2 using cells expressing the three known members of the ITGA4/ITGA9 family: Tera-2 cells, expressing ITGA9-ITGB1; HT1080 cells, expressing ITGA4-ITGB1; and RPMI 8866 cells, expressing ITGA4-ITGB7, and little or no ITGB1 on the cell surface [36], [39]. Control experiments showed that cell adhesion levels were very low with no substrate or a negative control substrate, BSA (<5% of input cells remaining in the well), that cells adhered well to positive control substrates (∼70–85% of input cells remaining in the wells with fibronectin or laminin as substrates), and that chelation of divalent cations abolished cell adhesion (<5% of input cells adhering to fibronectin, laminin, or ADAM2 in the presence of 10 mM EDTA), in agreement with what is known about integrin-mediated adhesion. Tera-2 cells and HT1080 cells adhered to ADAM2 (Fig. 3A,B), in agreement with other studies of ITGA4-ITGB1-expressing and ITGA9-ITGB1-expressing cells [27]. RPMI 8866 cells also adhered to ADAM2 (Fig. 3C). Adhesion of all three cell types to ADAM2 was inhibited by the peptide corresponding to the ECD-containing adhesion-mediating motif in the ADAM2 disintegrin domain, but not by the control peptide, ECA (Fig. 3).

The involvement of ITGA4/ITGA9 integrins in ADAM2 adhesion was demonstrated through the side-by-side comparisons of the effects of inhibitory peptides and antibodies, although these experiments also revealed something unexpected. The ITGA4/ITGA9-blocking peptide MLD inhibited adhesion of all three cell types to ADAM2 (Fig. 3). The MLD peptide also effectively inhibits HT1080 cell adhesion to a known ITGA4-ITGB1 ligand, the CS-1 (connecting segment 1) region of fibronectin (data not shown). Adhesion of Tera-2 cells and HT1080 cells to ADAM2 was inhibited by an anti-ITGA9 or anti-ITGA4 function-blocking antibody respectively; the extent of the antibody-mediated inhibition was similar to the extent of inhibition observed with the ECD or MLD peptides (Fig. 3A, B). Surprisingly, the anti-ITGA4 function-blocking antibody only partially inhibited the adhesion of RPMI 8866 cells to ADAM2, not as extensive as the inhibition with the ECD or MLD peptides (Fig. 3C). This differed from other work showing that RPMI 8866 cell adhesion to various substrates is inhibited by anti-ITGA4 antibodies; anti-ITGB7 antibodies have similar significant inhibitory effects whereas anti-ITGB1 antibodies do not affect RPMI 8866 cell adhesion to numerous substrates, including ADAMs [35], [36], [39]. Interestingly, the anti-ITGA9 function-blocking antibody inhibited RPMI 8866 cell adhesion to ADAM2 to a similar partial extent as the anti-ITGA4 antibody (60% and 67%; Fig. 3C). The combination of anti-ITGA9 and anti-ITGA4 antibodies inhibited adhesion to a similar extent as the ECD or MLD peptides (88%), as did an anti-ITGB7 function-blocking antibody (86%; Fig. 3C).

**Figure 4:**
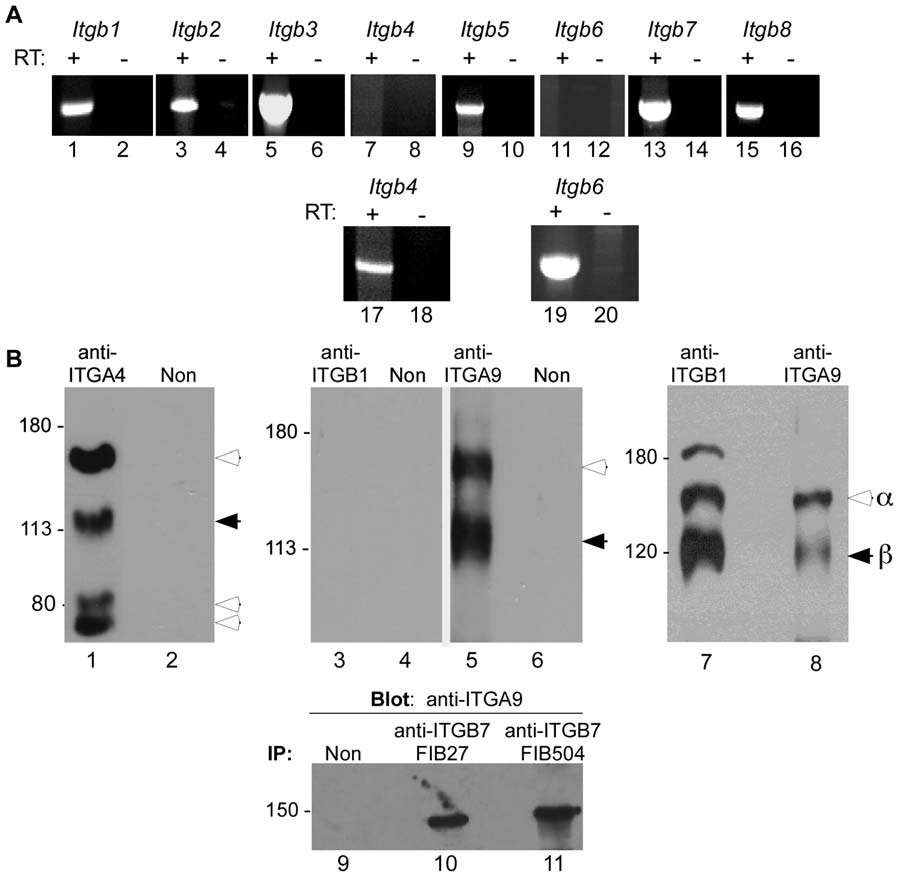
Integrin subunit expression in RPMI 8866 cells. **Panel A**. RT-PCR analysis of *ITGB* subunit mRNA expression in RPMI 8866 cells (lanes 1–16) or control tissue (spleen, lanes 17–20). First strand cDNA was prepared from RNA with reverse transcriptase (+), or, as a negative control, without RT (−). This shows that RPMI 8866 cells express *ITGB1*, *ITGB2*, *ITGB3*, *ITGB5*, *ITGB7*, and *ITGB8* mRNA. *ITGB4* and *ITGB6* were not detected in RPMI 8866 cells, but these PCR products could be amplified from spleen cDNA. **Panel B**. Lanes 1–6 show immunoprecipitations performed with lysates from surface-biotinylated RPMI 8866 cells, lanes 7–8 show immunoprecipitations performed with lysates from surface-biotinylated Tera-2 cells, and lanes 9–11 show immunoprecipitations performed with lysates from unlabeled RPMI 8866 cells; samples were run under non-reducing conditions. Immunoprecipitations were performed with anti-ITGA4 (lane 1; PS/2), anti-ITGB1 (lanes 3 and 7, ABII2), anti-ITGA9 (lanes 5 and 8, Y9A2), anti-ITGB7 (lane 10, FIB27; lane 11, FIB504) or appropriate species-matched nonimmune IgGs. Bands in blots with lanes 1–8 were detected with avidin, and the blot with lanes 9–11were probed by anti-ITGA9 immunoblotting. Open arrowheads indicate integrin α subunits, and solid arrowheads indicate integrin β subunits (Note: ITGA4 in Lane 1 is observed in intact and cleaved forms [45], [94].)

These data from cell adhesion assays suggested that RPMI 8866 cells adhered to ADAM2 not only through ITGA4-ITGB1 (α_4_β_7_) but also through **ITGA9**-ITGB7 (**α_9_**β**_7_**). The surprising finding of ITGA4- and ITGA9-mediated cell adhesion to ADAM2 prompted us to examine integrin β subunit expression in RPMI 8866 cells, as ITGA9 has only been reported to dimerize with ITGB1. RT-PCR demonstrated that RPMI 8866 cells express *ITGB1*, *ITGB2*, *ITGB3*_3_, *ITGB5*, *ITGB7* and *ITGB8* mRNA (Fig. 4A). Although *ITGB1* mRNA was detected, we did not detect ITGB1 protein in immunoprecipitations from lysates of surface-labeled RPMI 8866 cells (Fig. 4B, lane 3), in agreement with others' experiments showing little or no ITGB1 surface expression [35], [36], [37], [38], [39]. The anti-ITGB1 antibody was used successfully in control immunoprecipitations from Tera-2 lysates, as was the anti-ITGA9 antibody (Fig. 4B; lanes 7–8). The detection of *ITGB1* mRNA by RT-PCR suggests that the absence of ITGB1 protein on the RPMI 8866 cell surface is due to a failure in translation, trafficking, and/or maintenance/stability. Immunoprecipitations from RPMI 8866 lysates with an anti-ITGA4 antibody immunoprecipitated ITGA4 and a β subunit migrating at ∼120,000, the M_r_ of ITGB7 subunit (Fig. 4B, lane 1), essentially identical to results obtained with a similar cell line that expresses ITGA4-ITGB7 and not ITGB1 [45]. The anti-ITGA9 antibody immunoprecipitated two bands, ITGA9 (M_r_ =  ∼142,000) and a second band at M_r_ =  ∼120,000, the same electrophoretic mobility as the β subunit co-immunoprecipitating with ITGA4, and differing from the M_r_'s of ITGB2, ITGB3, ITGB5, ITGB8, which are readily distinguishable from ITGB7 as they migrate at a lower M_r_ of ∼90,000–100,000 [45], [46], [47], [48], [49]. Additionally, immunoprecipitations performed with anti-ITGB7 antibodies contain a band similar to the size of ITGA9 (M_r_ =  ∼142,000) that cross-reacts with an anti-ITGA9 antibody in immunoblots (Fig. 4B, lanes 10, 11). Taken together, these data suggest ITGB7 can dimerize with ITGA9 in RPMI 8866 cells.

**Figure 5:**
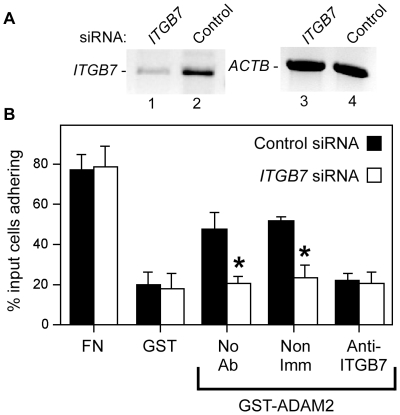
siRNA-mediated knockdown of *ITGB7* mRNA levels and effects on RPMI 8866 cell adhesion to ADAM2. **Panel A**: Sample results from an RT-PCR analysis of *ITGB7* siRNA-mediated knockdown. RNA was isolated from cells transfected with *ITGB7* siRNA (lanes 1, 3) or control siRNA (lanes 2, 4) at 48 hr post-transfection, first-strand cDNA was prepared, and primers specific for *ITGB7* or β-actin (*ACTB*) were used in PCR. (The levels of *ITGB7* knockdown observed in four replicate experiments were 100%, 85%, 83, and 39%.) **Panel B**: Cell adhesion assays were performed with the substrates plasma fibronectin (positive control), GST (negative control) or recombinant GST-ADAM2 with RPMI 8866 cells treated with control siRNA (solid bars) or *ITGB7* siRNA (open bars). *ITGB7*-siRNA-treated cells that were to be tested on GST-ADAM2 were either left untreated (No Ab) or were treated prior to being added to the wells with 20 µg/ml anti-ITGB7 antibody FIB27, or nonimmune rat IgG (Non IgG). The y-axes indicate the percentage of the input cells left adherent after washing; errors bars represent the SEM. Asterisks indicate p<0.05 as compared to control siRNA-treated cells.

Since RPMI 8866 cells express *Itgb1* mRNA (Fig. 4A), the possibility remained that low levels of ITGB1 on RPMI 8866 cell surfaces contribute adhesion to ADAM2. To confirm that RPMI 8866 cells used only ITGB7 integrins to adhere to ADAM2, we depleted RPMI 8866 cells of *ITGB7* by siRNA-mediated knockdown. The levels of *ITGB7* mRNA in RPMI 8866 cells treated with *ITGB7* siRNA were reduced to an average of 23% of the levels in cells treated with control non-targeting siRNA (n = 4 experiments; knockdown levels observed were 100%, 85%, 83, and 39%). Cells treated with control siRNA or with *ITGB7* siRNA adhered to fibronectin, and cells treated with control siRNA adhered to ADAM2. Knockdown of *ITGB7* reduced adhesion to ADAM2; *ITGB7*-knockdown cells had similar levels of adhesion to ADAM2 and to the negative control substrate (GST), indicating that the *ITGB7* siRNA treatment reduced adhesion to ADAM2 to baseline levels (Fig. 5). As an additional control, we assessed the effects of an anti- ITGB7 function-blocking antibody combined with *ITGB7* siRNA treatment (Fig. 5), to determine if residual levels of ITGB7 protein contribute to adhesion to ADAM2. These experiments revealed that adhesion to ADAM2 of cells treated with both *ITGB7* siRNA and the FIB27 anti-ITGB7 antibody was not different from cells treated with treated only with *ITGB7* siRNA. On the other hand, adhesion to ADAM2 of control siRNA-treated cells was inhibited by the FIB27 anti-ITGB7 antibody treatment. These results showed the siRNA-mediated knockdown in the *ITGB7* siRNA-treated cells was effective and that that there was little or no residual ITGB7 on the surface of *ITGB7* siRNA-treated cells contributing to adhesion to ADAM2.

## Discussion

This study had two goals, one specific, one more broad. We examined if ITGB1 (as the partner to ITGA9) functions as the primary integrin in mouse eggs that mediates egg interactions with ADAM2, and characterized the complement of integrin β subunits in mouse eggs. Second, since eggs in some mammalian species express other subunits of the ITGA4/ITGA9 family, we examined the interactions of ITGA4/ITGA9 integrins with ADAM2. This second set of experiments produced an unexpected result, indicating the presence of a novel integrin α-β combination in RPMI 8866 cells that functions as a binding partner for ADAM2. ITGA9 has only been reported to dimerize with ITGB1, and thus the ITGA9-ITGB7 heterodimer implicated in RPMI 8866 adhesion to ADAM2 is an unexpected integrin α-β subunit combination.

The mechanisms that drive integrin subunit dimerization are only partially characterized [50], [51], [52]. One factor that appears to affect integrin heterodimerization is subunit abundance in the cell. The relative abundance of a particular subunit can affect dimerization with partner subunits; for example, exogenous expression of an α subunit presents additional partners to pair with β subunits, affecting the α-β combinations expressed by a cell [53], [54], [55], [56], [57], [58]. In RPMI 8866 cells, the dimerization of both ITGA4 and ITGA9 with ITGB7 could be facilitated by the presence/abundance of ITGB7, combined with absence or very low abundance of ITGB1 (Fig. 4 and [36], [39]). In many cell types (including mouse eggs), ITGB1 is likely to be one of the most, if not the most, abundant β subunit, favoring ITGB1 dimerization with available α subunit(s) and likely also minimizing the chance for α subunits to dimerize with other β subunits besides ITGB1. Because of the contribution of subunit abundance to subunit dimerization, ITGA9-ITGB1 is likely to be the main ITGA9 integrin in many cells, including mouse eggs. We expect that RPMI 8866 cells are reflective of a small subset of cells that express little or no ITGB1 on the cell surface. Nevertheless, these data show that ITGA9 can dimerize with a β subunit other than ITGB1.

**Figure 6:**
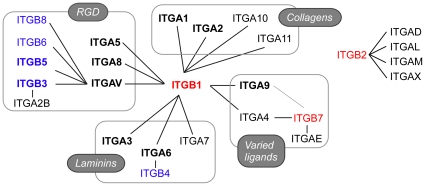
Heterodimer combinations of α/ITGA and β/ITGB subunits. Diagram is adapted from [1]. The three β subunits that are members of Vertebrate A branch or β_1_ family (ITGB1, ITGB2, ITGB7) are shown in red, and members of the Vertebrate B branch or β_3_ family (ITGB3, ITGB4, ITGB5, ITGB6, and ITGB8) are shown in blue [4], [5]. Ligands that are recognized by certain families of integrins are indicated in ovals. The ITGB2 integrins of leukocytes are shown to the right. Bolded subunits are detected in mouse oocytes.

The data in Figure 2, with other reports of integrin subunit expression, indicate that mouse eggs express three of eight β subunits and eight of 18 α subunits (*Itgb1*, *Itgb3*, *Itgb5*, *Itga1*, *Itga2*, *Itga3*, *Itga5*, *Itga6*, *Itga8*, *Itga9*, *Itgav*). Thus, mouse eggs could express at least ten different integrins, with five in the family of integrins that bind RGD tripeptide in ligands (e.g., fibronectin, vitronectin), two in the laminin-binding family, two in the collagen-binding family, and ITGA9-ITGB1, which can interact with a variety of ligands (Fig. 6) [1], [2]. Several of these integrins, especially ITGA9-ITGB1, have been shown to interact with ADAMs [59], [60], [61]. *Itgb7* mRNA was not detected in oocytes, and the extent of inhibition of ADAM2 binding to eggs by a function-blocking anti-ITGB1 antibody suggests that ITGB1 integrins are the major integrins mediating ADAM2-egg interactions. The other β subunits detected in mouse oocytes are *Itgb3* and *Itgb5*, although it appears that ITGA9 dimerizes primarily or exclusively with ITGB1, based on immunoprecipitations of ITGA9 from mouse egg lysates [10].

The possibility exists, however, that the situation is different in eggs of other species. ITGA4 is not detected in mouse eggs [10], but there is evidence for ITGA4 expression in bovine, hamster, porcine and human eggs, as well as some evidence for ITGB7 expression in human eggs [12], [13], [14], [15]. In a similar vein, *Itgb2* mRNA was not detected in mouse eggs, but ITGB2 is detected in human eggs [15], [20], [62]. Interestingly, there is some evidence suggesting that different species' eggs may rely on different receptors to varying extents. An anti-ITGA6 function-blocking monoclonal antibody (GoH3) has been reported to have some [19] or no [23], [63] effect on mouse sperm-egg interactions (even though this antibody binds to ITGA6 on the mouse egg), but this same antibody has moderate to significant inhibitory effects on human sperm-egg interactions [20], [64]. Similarly, antibodies to the tetraspanin CD9 inhibit mouse and pig mouse sperm-egg interactions [65], [66], [67], while two different anti-CD9 antibodies had no effect on human sperm-egg fusion, but partial inhibition is observed with an antibody to the tetraspanin CD151 [64]. It is worth considering that species-to-species variation is possible, and thus the finding here of an ITGA9-ITGB7 heterodimer may be relevant to fertilization in some species.

The ITGA9-ITGB7 heterodimer raises the question of whether ITGA9 (or other α subunits) in an *Itgb1*-deficient cell could dimerize with unexpected β subunits — a situation roughly comparable to RPMI 8866 cells. Cases of integrin deficiency are phenotypically alleviated in multiple ways. Functional compensation is one, as multiple integrins and other adhesion molecules can serve as receptors for a given ligand [1], [2]; this occurs in some integrin-deficient cells but not all (e.g., [68], [69]). Up-regulation of compensatory subunits is also possible, although is not detected in all integrin-deficient cells (e.g., [69], [70]). The data presented here suggest that alternative α-β heterodimerizations could be another possible compensatory mechanism in certain situations. A computational study suggests that α-β transmembrane domain interactions were largely conserved for nearly all α-β combinations, the 24 known combinations as well as the 120 other combinations [71]. This raises the question of whether the failure to observe a particular α-β heterodimer is indicative that the α-β combination cannot form, or that the α-β combination is a rare occurrence in nature or in the cellular contexts that have been examined. One proposed explanation for the fact that only certain α-β combinations have been observed is that that α integrin subunits may have gained or lost the ability to form heterodimers with certain β subunits during the course of gene duplication [5]. The originally observed ITGA4/ITGA9 heterodimer combinations could have been viewed as evidence that ITGA4 gained the ability to dimerize with ITGB7 and/or ITGA9 lost the ability to dimerize with ITGB7 as *Itga4* and *Itga9* diverged. However, the data here provide the insight that both ITGA4 and ITGA9 can dimerize with ITGB7. Future experiments could shed light on if this will extend to other subunits. Molecular evolution studies suggest two families of vertebrate β subunits: the Vertebrate A or β_1_ family (*Itgb1*, *Itgb2*, *Itgb7;* Fig. 6, in red) and Vertebrate B or β_3_ family (*Itgb3*, *Itgb4*, *Itgb5*, *Itgb6*, and *Itgb8*; Fig. 6, in blue) [4], [5]. ITGAV and ITGA6 dimerize with β subunits in both groups (Fig. 6); to our knowledge the abilities/inabilities of other α subunits to dimerize with A/β_1_ and B/β_3_ subunits has not been definitively determined. This will be an interesting area of future investigation in integrin function and evolution. ITGB1 and ITGB7 are members of the Vertebrate A/β_1_ family; it remains to be determined if ITGA4 and ITGA9 have the ability to dimerize with Vertebrate B/β_3_ family members, or only dimerize with Vertebrate A/β_1_ family members. There are numerous interesting future directions for studies of integrin function and α-β heterodimerization, including focused studies (e.g., RNAi of *ITGA9* in RPMI 8866 cells) and broader research to gain insights into integrin actions in normal and pathological conditions, and whether “compensatory dimerization” occurs in other cellular contexts, similar to what occurs in RPMI 8866 cells with low ITGB1 expression.

It is worth noting that there are proteins other than integrins on eggs and ADAMs on sperm that participate in sperm-egg membrane interaction [72], [73]. Only two, the tetraspanin CD9 on the egg and the immunoglobulin superfamily member IZUMO1 on the sperm, have been shown in gene knockout studies to be essential for murine sperm-egg interaction. Other knockouts have less severe defects or multiple gamete function defects (e.g., [74]), and mouse eggs deficient in *Itga9* or *Itgb1* do differ from wild-type eggs in their ability to support sperm binding [10], [22]. Even though such defects may not translate to a complete failure of fertilization, these partial loss-of-function defects could be an underlying cause of subfertility. This may be significant in humans, considering mice ovulate multiple eggs in short estrus cycles versus humans ovulating one egg once every ∼28 days. A gamete function deficiency that has only modest effects in the mouse could produce a more significant effect on human fertility, particularly since the clinical definition of infertility is an inability to conceive after a year of unprotected intercourse.

Finally, extending a broader biomedical context, integrins and ADAMs are involved in numerous processes and disease states – myoblast fusion, leukocyte homing, cancer, inflammatory diseases, and respiratory diseases, to name just a few [2], [61], [75], [76], [77], [78], [79] – making both molecular families of significant interest. The ITGA4/ITGA9 integrin family interacts with several ADAMs [27], [33], [39], [80], [81]. The work here shows that this integrin family includes ITGA9-ITGB7, and that this α-β integrin subunit combination can serve as a binding partner for ADAM2 and likely other ADAMs as well. Although ITGA9-ITGB7 is not a widely expressed integrin, the findings presented here nevertheless suggest that this α-β combination can function as an ADAM binding partner in certain cellular contexts, providing new avenues to appreciate the functions of both integrins and ADAMs.

## Materials and Methods

### Antibodies and peptides

The antibodies used were: anti-ITGB1 monoclonal Armenian hamster antibody Hmβ_1_-1 [82] (Pharmingen/BD Biosciences); anti-ITGA4 rat monoclonal antibody PS/2 [83] (Chemicon, Temecula, CA and Millipore, Billerica, MA); anti-ITGA4 rat monoclonal antibody R1-2 [84] (Pharmingen/BD Biosciences; San Diego, CA); anti-ITGA9 mouse monoclonal antibody Y9A2 [85] (Chemicon/Millipore); anti-ITGB1 rat monoclonal antibody AIIB2 [86] (Developmental Studies Hybridoma Bank, University of Iowa); anti-ITGB7 rat monoclonal antibody FIB27 [87] (Pharmingen/BD Biosciences); anti-ITGB7 rat monoclonal antibody FIB504 [87] (Santa Cruz Biotechnology, Santa Cruz, CA). Nonimmune control antibodies used were rat IgG, mouse IgG, rabbit IgG, and Armenian hamster IgG (Jackson Immunoresearch, Sigma-Aldrich, or BD Biosciences).

Peptides were produced as fusion peptides with bacterial alkaline phosphatase (BAP-presented peptides) as previously described [11], [24]. The peptide sequence AQDECDVT (hereafter referred to as ECD) corresponds to the adhesion-mediating sequence in the ADAM2 disintegrin loop [24], [25]; the negative control for this peptide is AQDECAVT (hereafter referred to as ECA), and was shown to have minimal effects on ADAM2 binding to mouse eggs [11], [24], in agreement with structure-function studies of ADAM2 [24], [25]. The sequence KRAMLDGLNDY (hereafter referred to as MLD) corresponds to the disintegrin loop in the snake venom disintegrin subunits EC3B and EC6A; the disintegrins EC3 or EC6 or this MLD peptide disrupt adhesion mediated by ITGA4/ITGA9 integrins [40], [41], [42]. The negative control peptide for MLD was KRAMAAGLNDY (hereafter referred to as MAA). Purified BAP-presented peptides were extensively dialyzed against culture medium compatible buffer (109.5 mM NaCl, 4.7 mM KCl, 1.2 mM KH_2_PO_4_, 7 mM NaHCO_3_, 15 mM HEPES, pH 7.4), and then concentrated to at least 10 mg/ml (Microcon microconcentrators; Amicon, Beverly, MA).

### Cell lines and culture

RPMI 8866 cells (a human B-cell lymphoblastoid cell line; gift of Dr. Ron Bowditch, University of Oklahoma, Oklahoma City, OK; also available from Sigma-Aldrich, St. Louis, MO) were grown in suspension in RPMI 1640 medium (Mediatech; Herden, VA), supplemented with 10% fetal bovine serum (FBS), 10 mM HEPES (Mediatech), 1 mM sodium pyruvate (Mediatech), 1 mM L-glutamine (Mediatech) and 50 µg/ml gentamicin (Cellgro, Herden, VA). Cells were cultured in a humidified incubator with 5% CO_2_ in air at 37°C and routinely split at the ratio of 1∶5 by diluting the cell suspension with the appropriate medium. Tera-2 cells (a human teratocarcinoma cell line; American Type Culture Collection, Manassas, VA) and HT1080 cells (a human fibrosarcoma cell line; American Type Culture Collection) were maintained as adherent cells in Dulbecco's Modified Eagle's medium (DMEM; Mediatech) supplemented with 10% fetal bovine serum (Sigma) and 50 µg/ml gentamicin. These cells were cultured in a humidified incubator with 5% CO_2_ in air at 37°C, and split when they neared confluency.

### Assays of ADAM2 interaction with cells

Recombinant mouse ADAM2 (fertilin β) disintegrin domain was generated as a fusion protein with glutathione S-transferase (GST) as previously described [11]. ADAM2 was cleaved from GST as previously described [11], or left intact as GST-ADAM2, with GST included as a negative control.

The binding of ADAM2 to zona pellucida (ZP)-free eggs was assessed as previously described [11]. In brief, ovulated mature, metaphase II eggs were collected from 6–8 week-old superovulated CF-1 mice (Harlan, Indianapolis, IN) at ∼13 hours after human chorionic gonadotropin injection, and cumulus cell and ZP removal was performed as previously described [10], [11]. ZP-free eggs were incubated in Whitten's medium containing 100 µM ECD, ECA, MLD or MAA peptides [11], or 100 µg/ml of anti-ITGB1 Hmβ_1_-1 antibody or 100 µg/ml nonimmune Armenian hamster IgG [10] for 60 min. The eggs were then incubated with 0.5 mg/ml recombinant ADAM2 in the presence of the indicated peptide or antibody for an additional 60 min. Egg-associated ADAM2 was measured using a luminometric immunoassay as previously described [11]. All work involving animals was conducted with approval from the Johns Hopkins University Animal Care and Use Committee.

Cell adhesion assays followed established protocols [88]. Immulon-2 HB 96-well plates (Dynatech Laboratories, Chantilly, CA) were coated with 75 µl of ADAM2 at 4°C overnight. Preliminary experiments of adhesion to ADAM2 using 25–200 µg/ml per well revealed that maximal adhesion was observed with 50 µg/ml, with similar levels of adhesion in wells coated with 100 or 200 µg/ml (X. Zhu, Ph.D. dissertation); studies here used ADAM2 and GST at 50 or 100 µg/ml. Prior to the adhesion assay, each well was blocked with 1% heat-inactivated (15 min, 80°C) BSA (Fraction V, Sigma) at 37°C for 1 hr. RPMI 8866 cells grown in suspension were collected by centrifugation at 1000× g for 5 min at room temperature, washed twice with serum-free RPMI 1640 or DMEM medium and resuspended to a concentration of 10^6^ cells/ml. For preparation of Tera-2 cells and HT1080 cells for adhesion assays, cells were detached by treatment with 0.025% trypsin-EDTA (Gibco, Invitrogen, Grand Island, NY), and washed twice in serum-free DMEM, then resuspended to a concentration of 10^6^ cells/ml in DMEM. In experiments in which the effects of anti-integrin antibodies or inhibitory peptides were examined, the detached cells were incubated with the indicated antibody (25–50 µg/ml as noted) or peptide (100 µM) for 30 min at 37°C prior to use in the adhesion assay. For the adhesion assay, 100 µl (10^5^ cells) were added to each well; cells were then cultured at 37°C for 1 hr. Following this incubation, unbound cells were washed off by rinsing each well three times with PBS (137 mM NaCl, 3 mM KCl, 8 mM Na_2_HPO_4_, 1.5 mM KH_2_PO_4_, pH 7.4). Adherent cells were fixed with 5% gluteraldehyde (Sigma) in PBS for 1 hr at room temperature and then stained with 0.1% crystal violet (Sigma) in PBS for 1 hr at room temperature. The fixed and stained cells were solubilized with 10% acetic acid in H_2_O for 30 min in room temperature. The optimal density of each well at 575 nm (OD_575_) was then read with a microplate reader (Molecular Device Corp, Sunnyvale, CA).

### Isolation of total RNA and reverse transcription (RT)-PCR

Oocyte RNA was prepared as previously described [10]. RNA was isolated from ∼10^7^ cultured cells or from mouse tissues (∼0.75 cm^3^ piece of mouse spleen or liver) with Trizol reagent (Invitrogen, Carlsbad, CA) according to manufacturer's instructions. First strand cDNA was synthesized from 1–5 µg total RNA with random primers using SuperScript Reverse Transcriptase III (Invitrogen) according to the manufacturer's recommendations. Primers used for PCR are shown in Table 1; primers match both human and mouse integrin subunits. PCR was conducted using Taq polymerase (New England BioLabs, Ipswich, MA) and a Thermo Electron Corporation Px2 Thermal Cycler (Milford, MA). PCR products were separated on a 1% agarose gel and were visualized under UV light after staining with ethidium bromide.

**Table 1:** Primers used for PCRof mouse and human integrin subunits.

Target	Forward (5′-3′)	Reverse (5′-3′)
*Itgb1* (β_1_)	GAGGTTCAATTTGAAATTAGC	GGCTCTGCACTGAACACATTC
*Itgb2* (β_2_)	CTACTCCATGCTTGATGACC	TTCTCCTTCTCAAAGCGCCT
*Itgb3* (β_3_)	GGACATCTACTACTTGATGG	ACCGTGTCTCCAATCTTGAG
*Itgb4* (β_4_)	CCTCATGGACTTCTCCAACT	GGGACACTGGAAGTTGTCAT
*Itgb5* (β_5_)	TATGCACTAGTGGAAGTGCC	CCCTCACACTTCCTCTGACC
*Itgb6* (β_6_)	TCTGACATTGTTCAGATTGC	ACTTCCAGTTCCACCTCAGA
*Itgb7* (β_7_)	CAGCTCATCATGGATGCTTA	GAAGAAGAACAGCTGGTTGTC
*Itgb8* (β_8_)	GGATGGTGTGTTCAAGAGGA	GATTCTATTTCACCAGCAATGG
*Itga1* (α_1_)	GGATCAACTTTAGTCACCAA	TGTGAATAATGAGCACTGAA
*Itga8* (α_8_)	GCCCAGCTTCTGCTGCACCG	CCCAAGGTCACACACCACCA
*Itgad* (α_D_)	TGGATCTCGACTCGTGGTGG	CACTTTTTCGGGCCCCATTC
*Actb* (β-actin)	CCAACTGGGACGACATGGAG	CCTGCTTGCTGATCCACATC

### Cell surface biotinylation, immunoprecipitation and immunoblotting

About 1–3×10^7^ cells were collected from one T-75 cm^2^ tissue culture flask (BD Falcon; Franklin Lakes, NJ), pelleted by centrifugation at 1500× g for 5 min and washed twice with PBS. The pelleted cells were resuspended in 1 ml serum-free medium (DMEM or RPMI 1640) containing 0.5 mg/ml EZ-link sulfo-LC-biotin (Pierce Chemical Company, Rockford, IL) and incubated at 37°C for 30 minutes with constant rotation. The reaction was quenched with by adding 10 µl of 1 M Tris-HCl (pH 7.5), after which the cells were pelleted and washed with PBS to remove the remaining free biotin. The surface-biotinylated cells were lysed in 500 µl lysis buffer (50 mM Tris-HCl, pH 7.4, 150 mM NaCl, 2 mM CaCl_2_ and 1% Triton-X-100) supplemented with 1 mg/ml leupeptin, 1 mg/ml aprotinin and 1 mM AESBF (Sigma) for 30 minutes on ice, followed by centrifugation at 14,000× g for 15 min at 4°C. Total protein concentration of the resulting lysate was determined by BCA Microassay kit (Pierce).

For immunoprecipitations, cell lysates were first pre-cleared by adding 40 µl Protein G-Sepharose beads (Upstate, Lake Placid, NY) to the lysate (∼40 µg total protein) and mixed at 4°C overnight. The precleared lysates were then combined with 1 µg of the appropriate anti-integrin antibody or nonimmune IgGs and incubated at 4°C for 4 hr with constant rotation. The antigen-antibody complexes were captured onto 20 µl Protein G beads by tumbling at 4°C overnight. The Protein G-Sepharose beads bound with integrin-antibody complexes were washed five times with ice-cold lysis buffer at 4°C. Following washing, the beads were resuspended in SDS-PAGE sample buffer (2% SDS, 5.5% sucrose, 0.006% bromophenol blue, 80 mM Tris-HCl, pH 6.8), heated at 100°C for 5 min; protein samples were separated on a 7% non-reducing SDS-PAGE gel, then transferred to an Immobilon-P membrane (Millipore, Billerica, MA). Biotinylated proteins were detected with ImmunoPure ABC Peroxidase Staining Kit (Pierce). For Figure 3, lanes 9–11, immunoprecipitations were performed using nonimmune or anti-ITGB7 antibodies, and detection was performed with an anti-ITGA9 polyclonal antibody [89]. Although there is a report that this anti-ITGA9 antibody showed cross-reactivity in immunoblots of rat tissues, particularly with a M_r_ =  ∼100,000 band [90], this anti-ITGA9 antibody has not shown such cross-reactivity in immunoprecipitations from mouse or human lysates [89], [91], [92], [93]. The band that we detect here is M_r_ =  ∼142,000, in agreement with the expected electrophoretic mobility of ITGA9.

### siRNA-mediated knockdown of *ITGB7* in RPMI 8866 cells

siRNA studies used ON-TARGETplus Duplex J-008013-06-0020 corresponding to human *ITGB7* with sense sequence 5′ CCACAUUUCUUACGAAUCCUU 3′ and antisense sequence 5′ PGGAUUCGUAAGAAAUGUGGUU, and ON-TARGETplus siCONTROL non-targeting siRNA served as a negative control (Dharmacon, Lafayette, CO). RPMI 8866 cells were transfected with *ITGB7* or control siRNA according to the manufacturer's protocol. Briefly, 10^7^ cells were suspended in 400 µl serum-free RPMI 1640 medium containing 1 µM *ITGB7* or control siRNA and 8 µg pMACS K^k^.II plasmid (Miltenyi Biotec Inc., Auburn, CA) in an electroporation cuvette (BTX, 2 mm gap, Holliston, MA) and incubated in room temperature for 10 minutes. Cells were then electroporated at 270 V, 5 pulses, 10 msec each (BTX ECM® 830 electroporator, San Diego, CA) and immediately placed in a humidified incubator with 5% CO_2_ in air at 37°C for 10 minutes. Cells were then transferred into a T-75 cm^2^ tissue culture flask (Falcon) containing 10 ml pre-warmed 10% fetal bovine serum-supplemented culture medium and cultured for 48 hr. pMACS K^k^.II-transfected cells were enriched using a magnetic bead-tagged antibody to human H2Kk and a MACS apparatus (Miltenyi Biotec Inc.) according to the manufacturer's specifications. For cell adhesion assays, cells were eluted from MACS^©^ MS Separation column in serum-free RPMI 1640 medium. For assessment of RNA knockdown, RNA was isolated from ∼10^6^ cells transfected with either *ITBG7* or control siRNA. First strand cDNA was synthesized from 5 µg total RNA using random primers and SuperScript Reverse Transcriptase III (Invitrogen) according to the manufacturer's protocol. PCR primers for *ITGB7* and β-actin (*ACTB*) (Integrated DNA Technologies or Invitrogen) are shown in Table 1. For each set of primers, the linear range in semi-log plots of the amount of PCR product as a function of cycle number was determined; the cycle number used for each primer pair was in this linear range. After PCR, the products were separated and visualized with UV light as described above. The signal intensities of the bands were quantified using Quantity One version 4.4.1 software (Bio-Rad Laboratories, Hercules, CA). The *ITGB7* signal was expressed as a ratio to the signal intensity of the control *ACTB* bands.

### Statistical Analysis

Statistical analyses were performed by ANOVA with Fisher's protected least significance difference post-hoc testing, performed with StatView version 5.5 (SAS Institute, Cary, NC).
